# Pooling Nasopharyngeal Swab Specimens to Improve Testing Capacity for SARS-CoV-2 by Real-Time RT-PCR

**DOI:** 10.1186/s12575-021-00156-6

**Published:** 2021-09-30

**Authors:** Imene Handous, Naila Hannachi, Manel Marzouk, Olfa Hazgui, Nissaf Bouafif Ep Ben Alaya, Jalel Boukadida

**Affiliations:** 1 Laboratoire de Microbiologie, UR12SP34, Hôpital Farhat Hached, Université de Sousse, Faculté de Médecine de Sousse, Sousse, 4000 Tunisie; 2 Université de Sfax, Ecole Nationale d’Ingénieurs de Sfax, Sfax, 3038 Tunisie; 3 Ministère de la Santé, Observatoire national des maladies nouvelles et émergentes, 1006 Tunis, Tunisie

**Keywords:** SARS-CoV-2, COVID-19, RT-qPCR, Pooling, Nasopharyngeal

## Abstract

**Background:**

The detection of SARS-CoV-2 using qRT-PCR with the pooling of samples can reduce workload and costs especially when the prevalence rate of COVID-19 in a population is low.

To analyse the effect of pooling samples on the sensitivity of RT-qPCR for severe acute respiratory syndrome coronavirus 2 (SARS-CoV-2) detection, we compared the cycle threshold (Ct) values of pools of 5 and 10 that tested positive with Ct values of individual samples that tested positive in that pool. Twenty positive nasopharyngeal (NP) specimens with low to high viral load were selected and pooled individually with four and nine negative NP.

**Results:**

In NP specimens, the sensitivity of pools of 5 and 10 were 90 and 85%, compared to individual sample testing, respectively. The RT-qPCR sensitivity of pools of 5 and 10 against individual testing were not significantly different (*p* > 0.05). Detection of positive samples with low Ct values (< 36) was consistently achieved in pools of 5 and 10. However, there were higher false negatives when samples with high ct values (> 36) were pooled and tested. The mean C_t_ values obtained with the 5-sample pooled testing exceeded individual sample testing by 1.85 ± 1.09 cycles, while C_t_ values obtained with the 10-sample pooling exceeded individual sample testing by 3.4 ± 1.65 cycles.

**Conclusions:**

In a low prevalence setting, testing capacity can be increased by pooling 5 or 10 samples, but the risk of additional false negatives needs to be considered.

## Introduction

Coronavirus Disease 2019 (COVID-19) pandemic has caused the worldwide public health emergency, the most serious one for the past 100 years. The World Health Organization estimates on 9 October 2020 over 36 million infected individuals and close to 1,049,810 reported deaths worldwide (https://covid19.who.int/). After the highest peaks in Asiatic and European countries, come the turn of the American and the African continents. The African countries are hit hard by the Covid-19 pandemic for a number of reasons. One particular reason is the limited availability of sufficient reagents and PCR Kits for the diagnosis of SARS-CoV-2 infection. In time, COVID-19 testing is one of the most important tools used to contain the pandemic and break the chains of transmission. In developing countries like in Tunisia, the need to increase testing capacity and conserve reagents has become the main concern in testing. Testing specimens in pools has already been used in blood banks around the world to detect viruses that can transmit blood transfusions, such as human immunodeficiency virus (HIV), hepatitis B and C viruses, and even in investigating the influenza pandemic (H1N1) of 2009 [1–3]. In the United States, pooling was used retrospectively to screen COVID-19 in specimens in the early period of the pandemic when viral circulation was low and this is because of its simplicity and cost-effectiveness [4].

Pooled specimens before extraction result in a significant reduction in the number of tests performed and would be more economical than individually testing especially by asymptomatic individuals. Successful application of pooling specimens depends upon knowledge of the limit of detection, sensitivity, and specificity of the assay, and the prevalence rate of the disease. Therefore, our main goal was to evaluate the efficiency of specimen pooling for testing of SARS-CoV-2 virus using the commercial kit “genesig real-Time PCR COVID-19.

## Materials and Methods

The study was approved by the ethics committee and medical research of the Farhat Hached University Hospital, Sousse, Tunisia (Reference number IRB00008931). This study was an evaluation of laboratory techniques using conserved clinical specimens.

### Sample Pooling

Nasopharyngeal (NP) specimens used in this study had been collected as part of clinical care from patients under investigation for COVID-19 infection placed in 2 mL of universal viral transport medium and sent to the laboratory of Microbiology of the University Hospital of Farhat Hached of Sousse. A total of 50 specimens had been tested negative for SARS-Cov2 using the available emergency authorized qualitative real-time PCR assays. A total of 20 SARS-CoV-2-positive samples were used for pooling. The selected Ct value of individual SARS-CoV-2 positive samples was spanning a large range of Ct of less than 40. Specimens with Ct values between 26 and 36 were considered to have low RNA viral load, while those with Ct values lower than 26 were considered to have a high viral load. Specimens with Ct values higher than 36 were considered weakly positive. All specimens had been stored at − 80 °C. Each positive nasopharyngeal swab specimen (0.140 mL) was mixed with 4 and 9 negative nasopharyngeal swab samples (0.140 mL each), obtaining pools of final volume 0.7 mL and 1.4 mL respectively. Out of 40 mini-pools either pools of 5 (*n* = 20) or 10 (n = 20) samples were made, with each containing one of the positive NP specimens (140 mL) mixed with 4 or 9 negative specimens. These pools were then vortexed for 5–10 s before RNA extraction and PCR testing.

### RNA Extraction and Real-Time PCR Assay

RNA was extracted from 0.140 mL of each individual and pooled specimen using the QIAamp viral RNA mini kit in conjunction with a QIAcube (QIAGEN, Hilden, Germany) following the manufacturer’s instructions. PCR amplification was performed using the Primerdesign Ltd. COVID-19 genesig assay targeting the (ORF1ab) genome region on the Applied Biosystem 7500 Real-Time PCR System (Applied Biosystems, Foster City, CA, USA) according to the manufacturers’ instruction. PCR was performed in duplicate (replicates I and II) for each extracted specimen. RNA internal extraction control was used to identify PCR inhibition and to evaluate RNA extraction purity and integrity of the PCR run. The detection limit of ORF1ab was ≤0.33 copies/μL. To improve the sensitivity of the pooling method, the number of cycles has been increased to 50. According to the laboratory’s protocol, samples with Ct > 36 that tested weakly positive with only one of the replicates are defined as inconclusive and required confirmation by retesting.

### Statistical Analysis

Statistical analysis was performed using SPSS software (version 22.0). The cycle thresholds (Ct) for the amplification of the ORF1ab gene were analyzed. The results are expressed as the average (mean) ± standard deviation (SD). Statistical significance of mean Ct differences (ΔCt) among pools of 5 and 10 were detected by Student’s T-test. *P* < 0.05 was considered statistically significant.

## Results

Comparison of C_t_ between the original and pooled SARS-CoV-2 positive samples are detailed in Table 1. PCR inhibition was not observed in all the NP specimens used in the present study and minimal variations were observed in all the internal control C_T_ values (data not shown). Positive pools of 5 and 10 samples could still be confidently identified, even if Ct values of single samples were up to 36. In pooled testing of 5 samples (Ct > 36), only two low positive specimens have been determined inconclusive and when tested in a pool of 10 samples, one positive specimen has been determined to be inconclusive, and two positive specimens have been determined to be undetected for both replicates. The overall sensitivity of the pool testing approach, with 5 and 10 specimens per pool, were 90 and 85% compared to individual tests. The sensitivity, of RT-qPCR pool-5 and pool-10 against individual testing, were not significantly different (*p* > 0.05). Our results showed that mean Ct values of the ORF1ab gene in both positive pools of 5 and 10 were 33.56 ± 4.1 and 34.9 ± 4.34 respectively, significantly higher than that of the un-pooled samples (*p* = 0.03, *p* = 0.001, respectively), indicating reduced viral loads. For these samples, the mean Ct values differences (ΔCt) between individual positive samples and pooled tests were 1.85 ± 1.09 in pools of 5, and 3.4 ± 1.65 in pools of 10. As expected, the mean Ct differences (ΔCt) between the original and each dilution aligned well with results expected for the given dilutions (i.e., In a real-time RT-PCR assay with 100% efficiency a 1:10 dilution leads to a 3.3 Ct value rise).

**Table 1:** Detection of SARS-CoV-19 RNA by RT-PCR in pooled nasopharyngeal (NP) specimens from patients with COVID-19

Specimen N°	Target	Nonpooled (Ct)			Positive NP specimen pooled with four negative NP specimens (Ct)				Positive NP specimen pooled with nine negative NP specimens (Ct)			
		Ct Replicate I	Ct Replicate II	Ct Average	Ct Replicate I	Ct Replicate II	Ct Average	Δ_**Ct**_	Ct Replicate I	Ct Replicate II	Ct Average	Δ_**Ct**_
**1**	**SARS-CoV-2 (orf1ab)**	17.85	18.79	18.32	20.59	21.15	20.87	2.55	21.32	22.03	21.67	3.35
**2**		27.35	26.57	26.96	31.27	30.63	30	3.03	31.35	30.75	31.05	4.09
**3**		27.39	27.24	27.32	28.58	28.75	28.67	1.35	29.34	29.30	29.32	2
**4**		28.85	26	27.42	31.53	29.71	30.62	3.2	32.50	32.64	32.57	5.15
**5**		29.46	29.61	29.53	31.47	31.55	31.52	1.93	34.74	34.65	34.7	5.16
**6**		30	33.06	31.53	33.8	33.2	33.5	2.46	36.20	36.32	36.26	4.77
**7**		32.18	32.54	32.36	35.15	34.85	35	2.63	35.76	36.66	36.21	3.8
**8**		32.56	32.17	32.37	35.82	35.09	35.45	3.08	35.89	36.63	36.26	3.89
**9**		32	33.36	32.68	36.7	36.64	36.67	3.99	39.43	38.91	39.17	6.49
**10**		32.15	33.37	32.76	31.9	32.92	32.44	0.32	32.61	33.84	33.22	0.46
**11**		33.49	33.12	33.30	36.85	33.15	35	1.7	37.09	37.31	36.56	3.26
**12**		32.7	34.22	33.46	35.19	34.98	35.08	1.62	37.27	37.74	37.50	4.4
**13**		33.62	33.41	33.52	37.27	35.39	36.33	2.81	37.32	38	37.66	4.14
**14**		35.23	35.61	35.23	35.51	37.29	36.4	1.16	37.2	38.51	37.85	2.62
**15**		35.74	35.80	35.77	36.26	36	36.13	0.36	37.46	37.89	37.68	1.91
**16**		36.52	35.52	36.03	35.63	37.33	36.48	0.45	36.87	37.99	37.43	0.56
**17**		36.39	35.98	36.18	36.72	36.6	36.66	0.48	37.85	38.23	38.03	1.85
**18**		36.20	36.33	36.22	37.47	37.05	37.26	1.04	41.1	ND	ND	–
**19**		37.05	36.58	36.81	37.07	ND	–	–	ND	ND	ND	–
**20**		38.0	38.32	38.16	38.73	ND	–	–	ND	ND	ND	–
	**Mean Δ**_**Ct**_ **± SD**			32.29 ± 4.61			33.56 ± 4.1	1.85 ± 1.09			34.9 ± 4.34	3.4 ± 1.65

*Ct* Cycle Threshold, *ND* Not detected, *NP* Nasopharyngeal, *Δ*_*Ct* Ct individual-Ct pooled_, *SD* Standard Deviation

## Discussion

Due to the huge number of tests that are being carried out universally, reagents needed for the SARS-CoV-2 testing are in short supply to ensure the rapid diagnostic process in patients with COVID-19 [5, 6]. Pooling specimens have been shown to increase testing capacity and reduce the cost per test for the detection of viral infections such as HIV, hepatitis B and C virus [1, 3]**.** A key step to control the epidemics is to implement fast and sensitive approaches for diagnostic. In the current study, pooling specimens were used to detect SARS-CoV-2 in NP specimens using the Primerdesign Ltd. COVID-19 genesig assay. Our data suggest that pooling of 5 or 10 samples per pool can increase test capacity with the existing equipment and test kits and detects positive samples with sufficient diagnostic accuracy. In contrast to developed countries, detailed published data on the practical use of pooled testing in outbreaks in resource-limited settings are lacking. There is only scarce information available in Tunisia [7]. The main limitations of the literature published so far are the small number of cases processed by this technique [8], mathematical models and the theoretical considerations [9, 10]. Three different approaches of pooling have been proposed: to pool swab samples from different patients during the collection process into a single volume of transport media (VTM), to pool the VTM of the samples from different samples to create a homogeneous pool in the laboratory and to pool the nucleic acid where an aliquot of RNA extracted from each sample is collected to create a homogeneous pool [11]. For laboratories, considering the positivity rate should be taken into consideration before implementing a pooling approach because pool testing is most efficient when applied in settings of low virus prevalence which may be more likely in an asymptomatic population especially in settings with limited capacity such in African countries. On the basis of our laboratory results, large cohorts and, testing on asymptomatic individuals can be carried out in particular in limited settings. The same as with previous studies when the incidence rate of SARS-CoV-2 infection is 10% or less, pool testing will result in the saving of reagents and personnel time with an increase in the testing capability of at least 69% [7, 12]. Pooling is not efficient during periods of high prevalence rate (>15%) when every pool yields a positive result and thus demand subsequent retesting to every specimen in the pool separately [1]. One strategy to avoid this is to adjust the size of the pool according to the infection prevalence. It is recommended that laboratories evaluate the pooling approach against the disease prevalence in the geographical area they serve before using this for routine diagnostic purposes.

The optimal number of pooled samples depends on the laboratory’s protocols and especially the limit of detection of the RT-PCR assay. Sensitivity for the various pool sizes can be improved with repeated sampling. The main disadvantage of pool testing is that there might be a decrease in test sensitivity. The sensitivity of the pooling is affected by many factors such as the sensitivity of the kit, the used dilution, collection sample techniques, samples type (NP, oropharyngeal, nasal, etc.), sample transport temperature, and viral load [13–15].

A recent study showed very encouraging results for SARS-CoV-2 using pools of up to 7 samples before the extraction and up to 60 samples after the RNA was extracted with false negativity of 10% [16], but this will mainly depend on validation protocol. Similar to our study, Wacharapluesadee et al. [17] used a pool size of 10 and found a 13.3% of false-negative due to positive samples with a low viral load. One of the major factors affecting the test sensitivity is the dilution effect of the pooled samples, leading to a higher rate of false negatives [18, 19]. The similar pooling strategy approach was presented by Sawicki, R et al. [8] who randomly pooled one positive sample and five negative individual NP swabs and one positive sample and eight negative individual samples, and who recommended concentration before the extraction step. The study results showed that pool testing could detect even up to a single positive sample with a Ct value as high as 34. In the current study, the detection of a specimen with a Ct value < 36 was not compromised when pooled with four or nine negative specimens. However, weak positive individual samples (Ct value > 36) might escape detection in pools. This is in line with recent works that reported a decreased sensitivity of pool testing at similar dilutions of pools of 5 and 10 in the presence of low viral load samples (Ct value > 36) [17–21]. Analysis of pool results requires close attention to inconclusive-result pools, as these may contain individual positive samples [1]. It is therefore advisable to test again a patient on whom there is a high clinical suspicion of COVID-19 infection even if the first test was negative. Weak positive samples were seen typically in convalescent patients after the resolution of symptoms [22]. These patients were not likely to be infectious and therefore were unlikely to be able to transmit the virus to another person [23–26]. In another study, no dilution errors were reported in very small pools (*n* = 5) [12]. Moreover, Yelin et al. [16] have concluded that the dilution effect is minimal for groups of 32 samples, but this is not a consistent finding in all studies. Similar to our study pools consisting of many samples may require additional amplification cycles due to lower viral load in the pooled samples [16, 27].

The dilution effect due to pooling was considered to be of varying importance among the studies, which is likely because, in the different experimental settings, samples with different viral loads were pooled. In this study, we found that the probability of detection of the positive pool decreased as the Ct value of the single positive sample in the pool increased (lower viral load). The mean Ct differences of the ORF1ab target of specimens when originally tested compared to undiluted specimens after one freeze-thaw was very small. On average, C_t_ values obtained with the 5-sample pooled testing exceeded individual sample testing by 1.85 ± 1.09 cycles while C_t_ values obtained with the 10-sample pooling exceeded individual sample testing by 3.4 ± 1.65 cycles. The viral load has been suggested as another important factor affecting the sensitivity of the assay in pooled samples. Further, test sensitivity of pooling depends on the distribution of viral loads across individuals and over the course of infection (growth versus decay) with implications for appropriate surveillance and the interpretation of a viral load from a sample pool [28, 29]. Despite the fast growth of the literature on theoretically optimal pooling designs for COVID-19 testing, formal inclusion of biological variation (i.e., viral loads) and incorporation of general position along the epidemic curve has received minimal attention [28–30]. Understanding the accurate performance of pooled testing requires modeling the prevalence in prolonged or multiple epidemic periods and the dynamics of viral load [29].

The results of our laboratory analyses should be interpreted with caution because conducted on a limited number of specimens. Additional studies need to be performed in order to determine if a larger input volume than that recommended for the QIAamp viral RNA mini kit will provide better results using this pooling protocol. The procedure evaluated herein was improved by increasing the number of RT-PCR cycles. However, it is possible that this approach can be further improved by using fresh specimens and by collecting samples in a smaller volume of transport medium, or by collecting samples directly in a small volume of lysis buffer provided that specimens are processed without delay [20].

Due to the global shortage of reagents and kits for SARS-CoV-2 (CoV-2) tests, the implementation of a single marker assay rises as a feasible and cost-effective option for diagnostics especially in low and middle-income countries. However, recent studies suggest that SARS-CoV-2 assay targeting two positions in the genome may improve the efficiency of the detection of positive specimens in low-size mini pools [20].

## Conclusion

According to the findings of this study, pooling samples for SARS-CoV-2 detection by real-time RT-PCR may be an acceptable method with minimal loss of sensitivity even for low virus loads**.** Pooling specimens will help to increase coronavirus testing capacity in order to meet the high demand for testing in the mass screening programs needed in the early identification and isolation of asymptomatic individuals especially during the constrained supply of reagents and PCR kits for the diagnosis of SARS-CoV-2 infection.

## Data Availability

The authors confirm that the data supporting the findings of this study are available within the article.

## Abbreviations

COVID-19: Coronavirus Disease 2019
Ct: Cycle threshold
NP: Nasopharyngeal
RT-qPCR: Reverse transcription real time PCR
RNA: Ribonucleic acid
SARS-CoV-2: Severe acute respiratory syndrome coronavirus 2
VTM: Volume of Transport Media
